# Supplementary material for: A Humanized Yeast Phenomic Model of Deoxycytidine Kinase to Predict Genetic Buffering of Nucleoside Analog Cytotoxicity
Source: Genes (Basel). 2019 Sep 30;10(10):770. doi: 10.3390/genes10100770 (PMC6826991; doi:10.3390/genes10100770)
Supplement: Supplementary file 1 [file genes-10-00770-s001.zip › Additional_Files/Additional_File7_GOTermHeatmaps.zip!/Additional_File7_GOTermHeatmaps/pyruvate_metabolic_process.pdf]

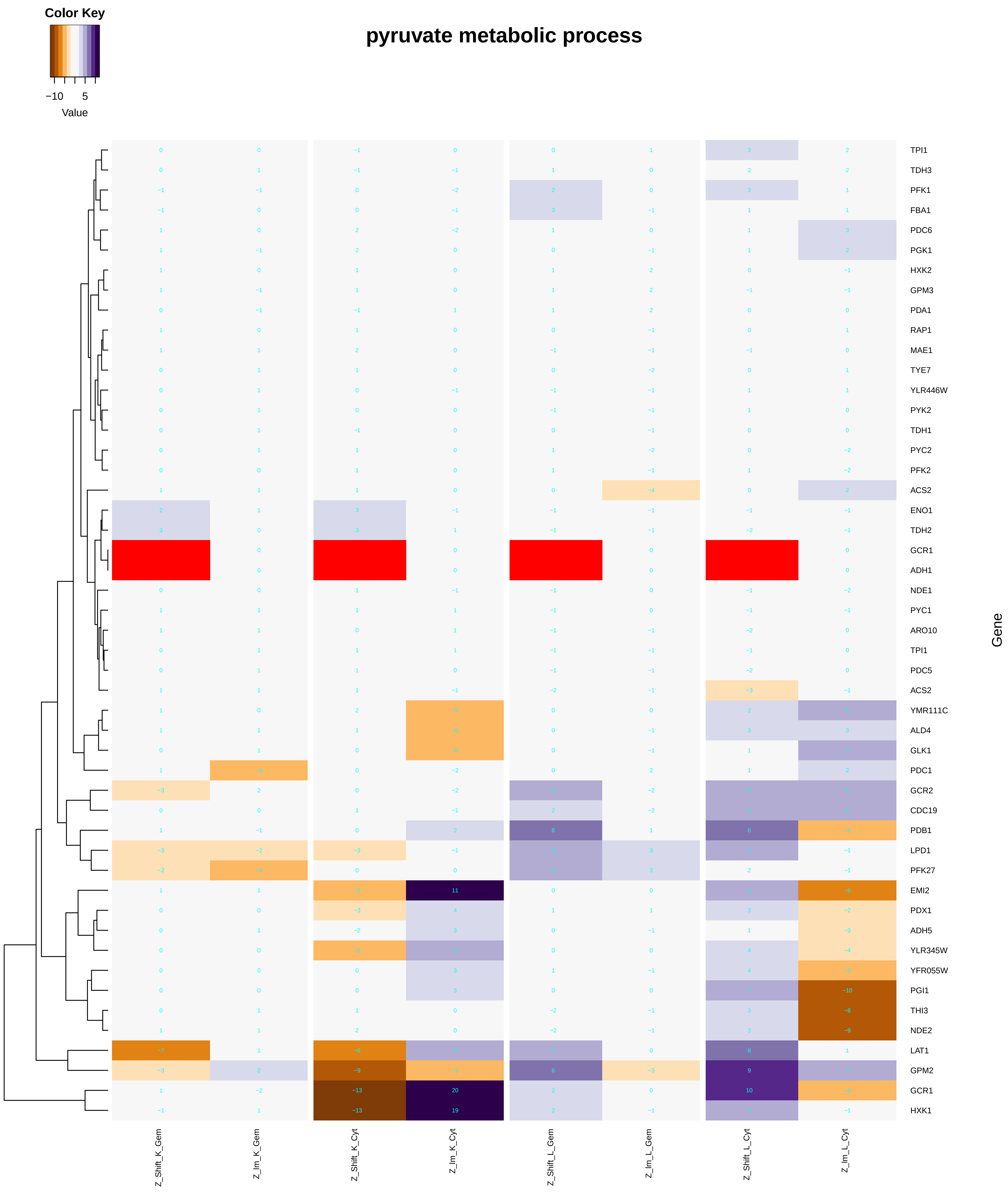
pyruvate metabolic process
Color Key
−10
5
Value
Gene
TPI1
TDH3
PFK1
FBA1
PDC6
PGK1
HXK2
GPM3
PDA1
RAP1
MAE1
TYE7
YLR446W
PYK2
TDH1
PYC2
PFK2
ACS2
ENO1
TDH2
GCR1
ADH1
NDE1
PYC1
ARO10
TPI1
PDC5
ACS2
YMR111C
ALD4
GLK1
PDC1
GCR2
CDC19
PDB1
LPD1
PFK27
EMI2
PDX1
ADH5
YLR345W
YFR055W
PGI1
THI3
NDE2
LAT1
GPM2
GCR1
HXK1
Z_Shift_K_Gem
Z_Im_K_Gem
Z_Shift_K_Cyt
Z_Im_K_Cyt
Z_Shift_L_Gem
Z_Im_L_Gem
Z_Shift_L_Cyt
Z_Im_L_Cyt

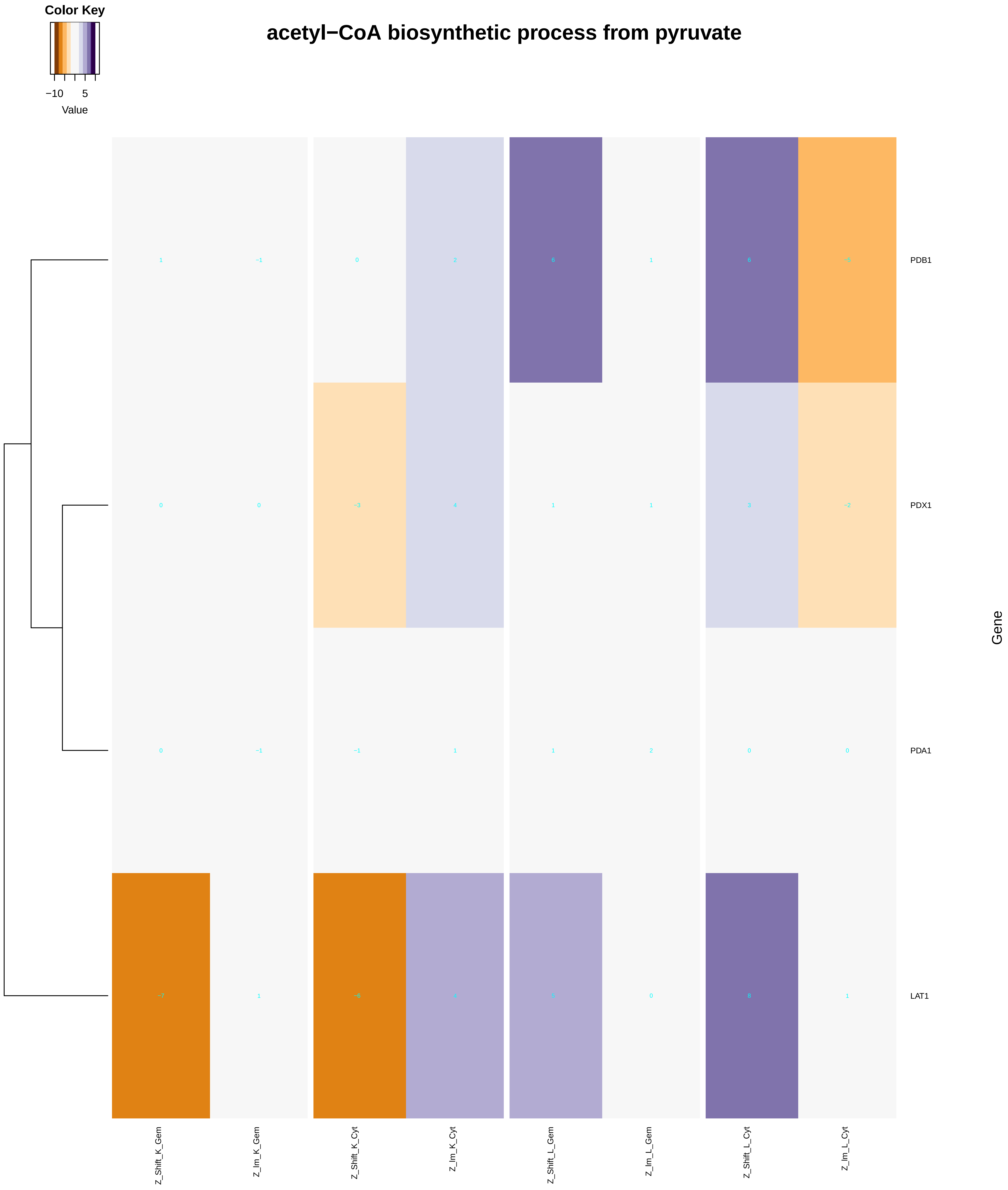

Color Key
−10
5
Value
acetyl−CoA biosynthetic process from pyruvate
PDB1
PDX1
PDA1
LAT1
Gene
Z_Shift_K_Gem
Z_Im_K_Gem
Z_Shift_K_Cyt
Z_Im_K_Cyt
Z_Shift_L_Gem
Z_Im_L_Gem
Z_Shift_L_Cyt
Z_Im_L_Cyt

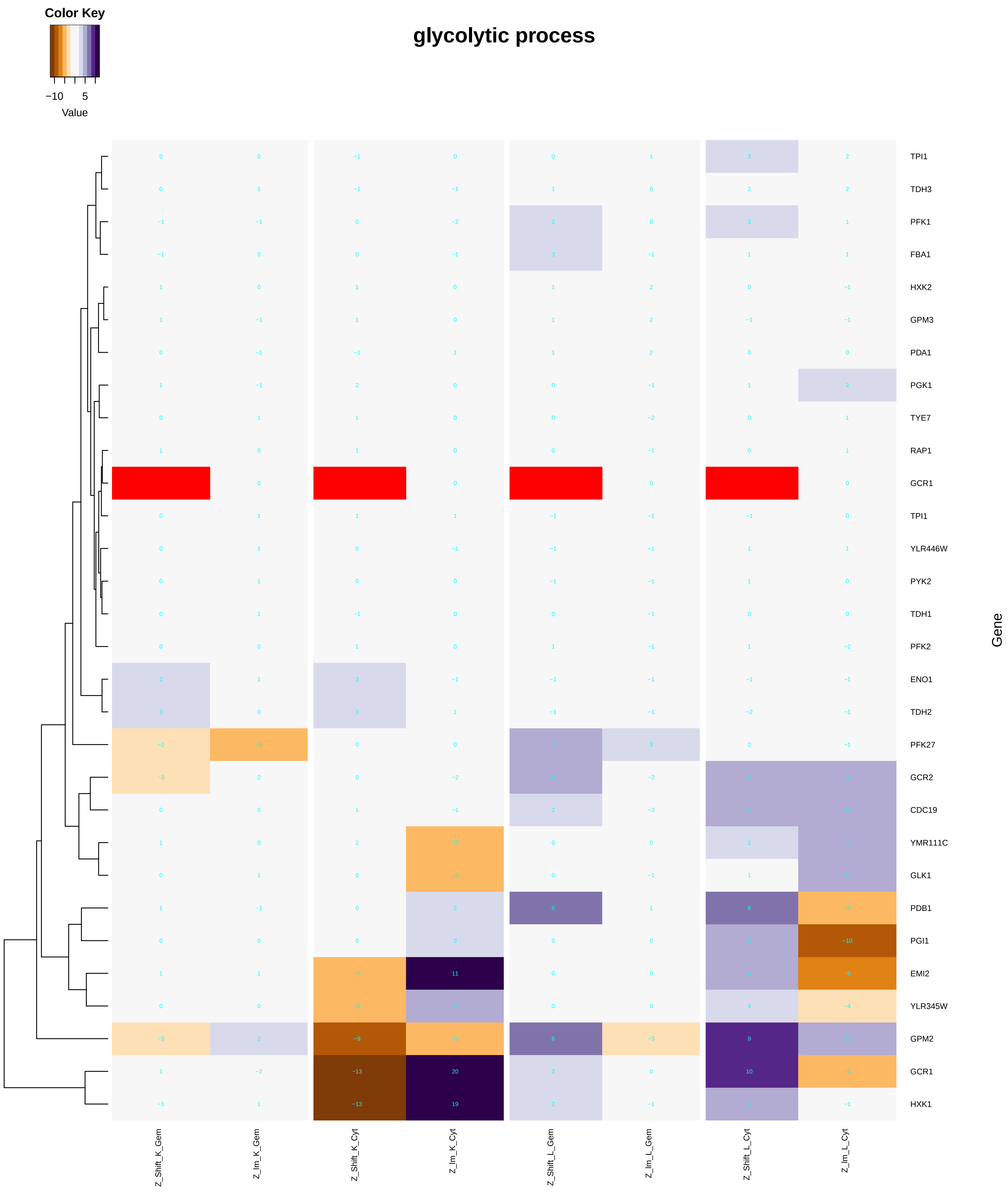

Color Key
−10
5
Value
glycolytic process
TPI1
TDH3
PFK1
FBA1
HXK2
GPM3
PDA1
PGK1
TYE7
RAP1
GCR1
TPI1
YLR446W
PYK2
TDH1
PFK2
ENO1
TDH2
PFK27
GCR2
CDC19
YMR111C
GLK1
PDB1
PGI1
EMI2
YLR345W
GPM2
GCR1
HXK1
Gene
Z_Shift_K_Gem
Z_Im_K_Gem
Z_Shift_K_Cyt
Z_Im_K_Cyt
Z_Shift_L_Gem
Z_Im_L_Gem
Z_Shift_L_Cyt
Z_Im_L_Cyt

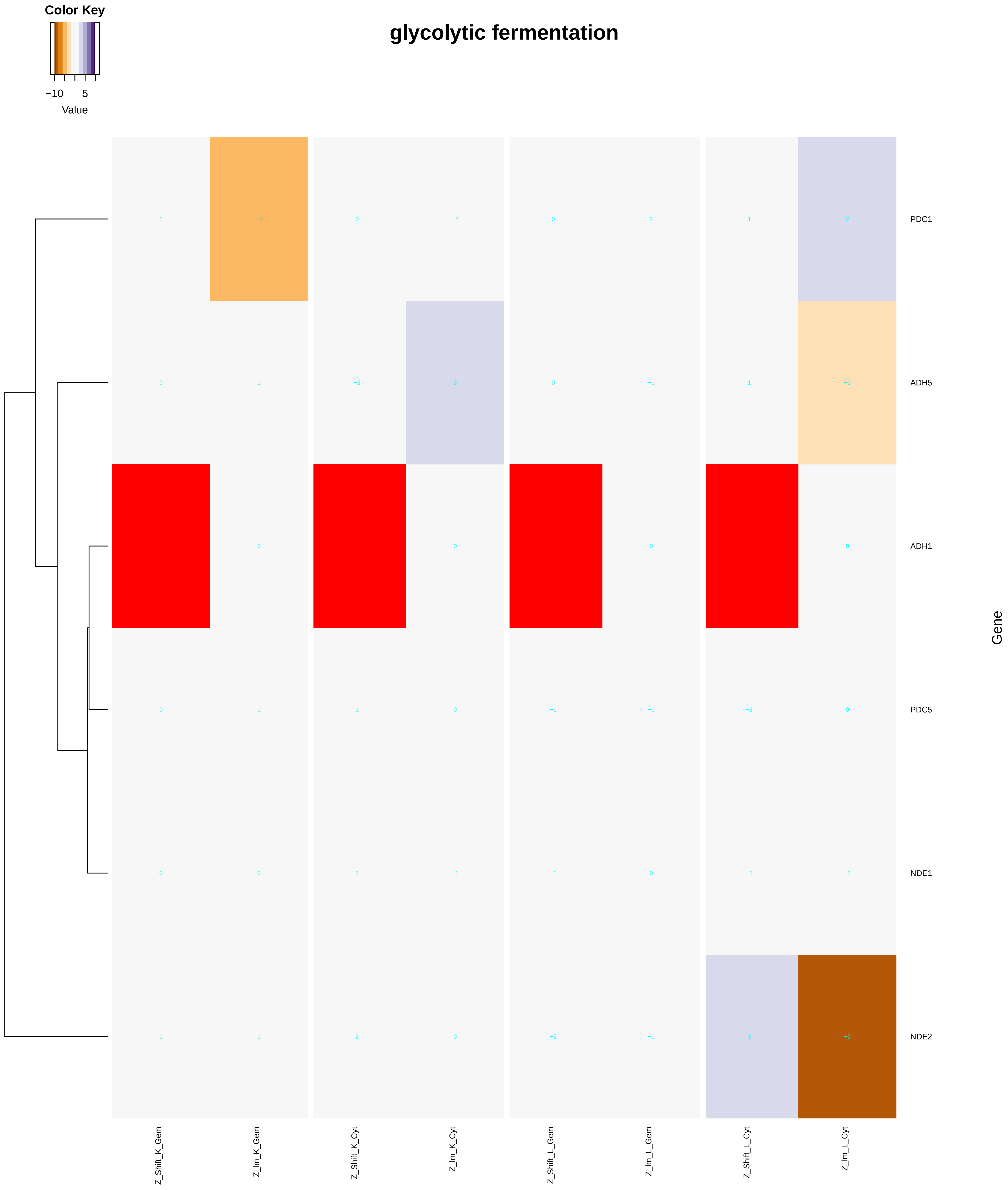
Color Key
−10
5
Value
glycolytic fermentation
PDC1
ADH5
ADH1
PDC5
NDE1
NDE2
Gene
Z_Shift_K_Gem
Z_Im_K_Gem
Z_Shift_K_Cyt
Z_Im_K_Cyt
Z_Shift_L_Gem
Z_Im_L_Gem
Z_Shift_L_Cyt
Z_Im_L_Cyt

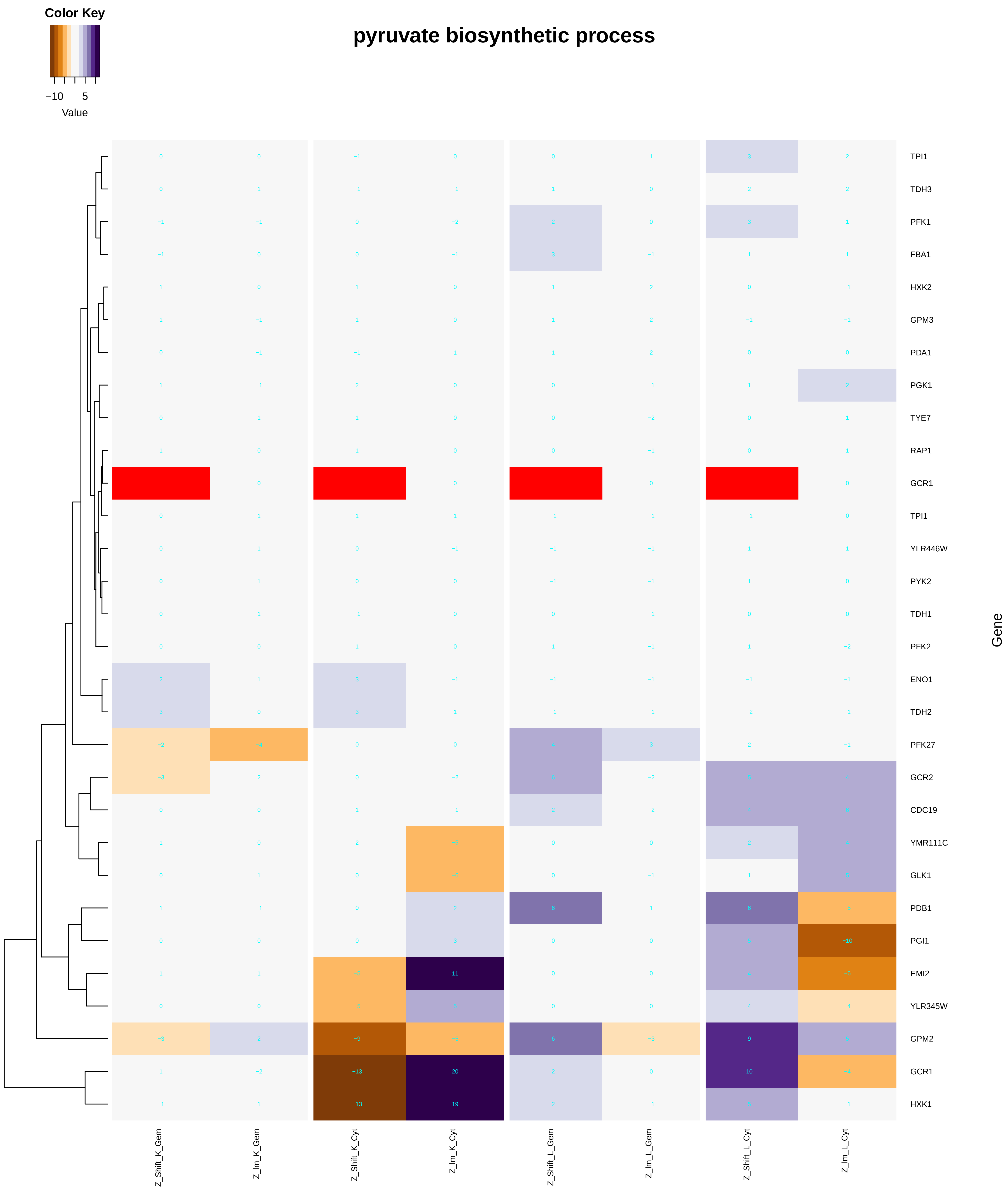
pyruvate biosynthetic process
Color Key
−10
5
Value
TPI1
TDH3
PFK1
FBA1
HXK2
GPM3
PDA1
PGK1
TYE7
RAP1
GCR1
TPI1
YLR446W
PYK2
TDH1
PFK2
ENO1
TDH2
PFK27
GCR2
CDC19
YMR111C
GLK1
PDB1
PGI1
EMI2
YLR345W
GPM2
GCR1
HXK1
Gene
Z_Shift_K_Gem
Z_Im_K_Gem
Z_Shift_K_Cyt
Z_Im_K_Cyt
Z_Shift_L_Gem
Z_Im_L_Gem
Z_Shift_L_Cyt
Z_Im_L_Cyt

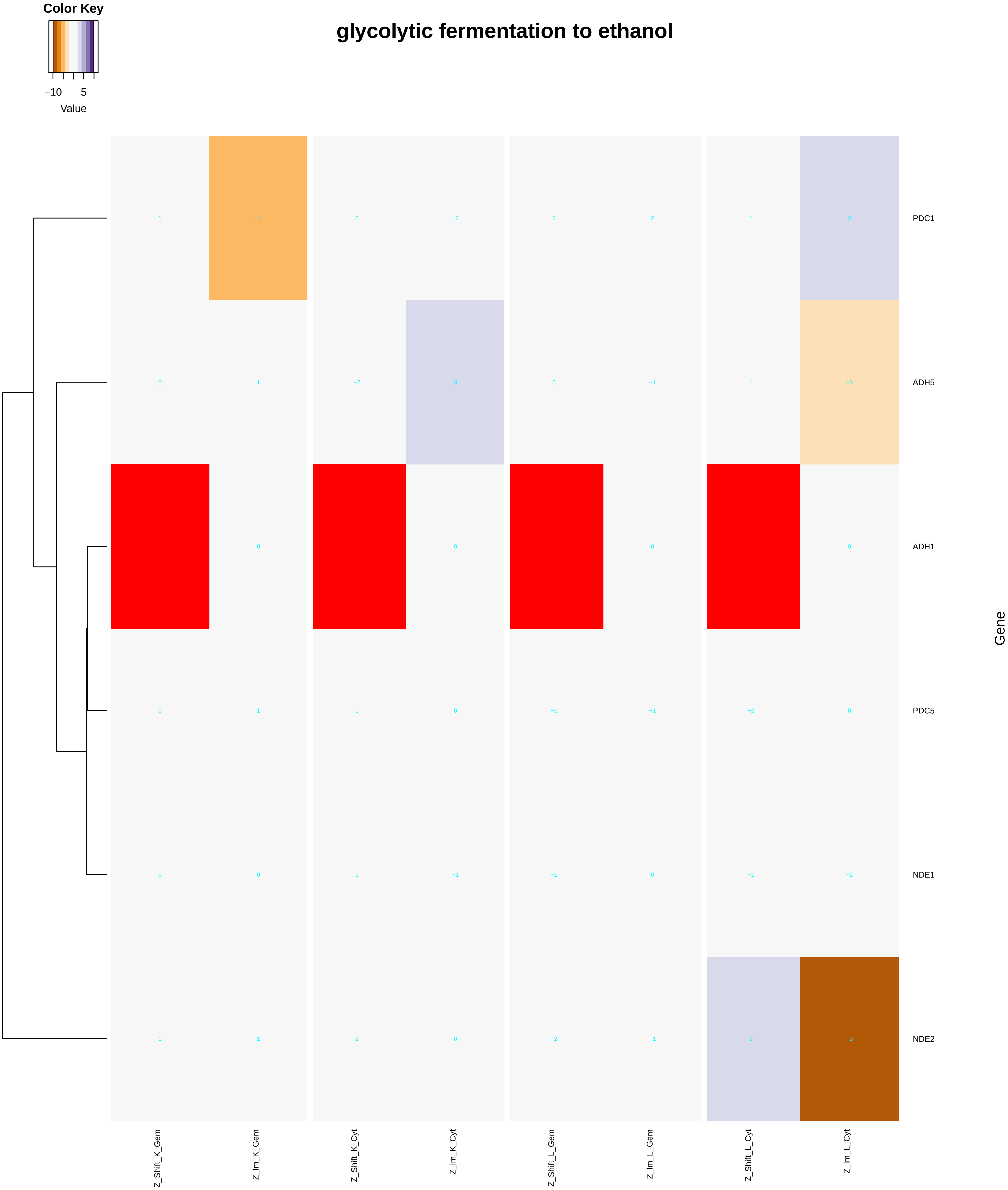

Color Key
−10
5
Value
glycolytic fermentation to ethanol
PDC1
1 −4 0 −2 0 2 1 2
ADH5
0 1 −2 3 0 −1 1 −3
ADH1
0 0 0 0
PDC5
0 1 1 0 −1 −1 −2 0
NDE1
0 0 1 −1 −1 0 −1 −2
NDE2
1 1 2 0 −2 −1 3 −9
Gene
Z_Shift_K_Gem
Z_Im_K_Gem
Z_Shift_K_Cyt
Z_Im_K_Cyt
Z_Shift_L_Gem
Z_Im_L_Gem
Z_Shift_L_Cyt
Z_Im_L_Cyt
